# ﻿A synopsis of *Christiana* DC. (Malvaceae, Brownlowioideae), with a new species from the Brazilian Atlantic Forest

**DOI:** 10.3897/phytokeys.253.145350

**Published:** 2025-02-28

**Authors:** Thales Silva Coutinho, Rafael G. Barbosa-Silva, Laurence J. Dorr

**Affiliations:** 1 Instituto de Biociências, Laboratório de Estudos Integrados de Plantas (LEIP), Universidade Federal de Mato Grosso, Cuiabá, Mato Grosso, 78060-900, Brazil Universidade Federal de Mato Grosso Cuiabá Brazil; 2 Coordenacão Botânica, Museu Paraense Emílio Goeldi, Belém, Pará, 66077-830, Brazil Museu Paraense Emílio Goeldi Belém Brazil; 3 Institute of Systematic Botany, New York Botanical Garden, 2900 Southern Boulevard, Bronx, NY, 10458-5126, USA New York Botanical Garden New York United States of America; 4 Department of Botany, MRC-166, National Museum of Natural History, Smithsonian Institution, P.O. Box 37012, Washington, D.C. 20013-7012, USA National Museum of Natural History Washington United States of America

**Keywords:** Endemism, Malvales, Restinga, taxonomy, Trans-Atlantic slave trade

## Abstract

A taxonomic synopsis is provided for the genus *Christiana*, with emphasis on the American species. Full synonymies, typifications, comments about distribution, and notes on main diagnostic characteristics, as well as conservation status and phenology, are provided. We describe a new species, *C.restingae* T.S. Cout., Barb.Silva & Dorr, **sp. nov**. With this addition, *Christiana* now comprises six species, with its center of richness in South America, where five species occur. The new species is endemic to Brazil where it is known only from Atlantic Forest and Restinga in the states of Espírito Santo and Rio de Janeiro, and is preliminarily assessed as Critically Endangered. Illustrations, images, a distribution map, SEM images of vegetative and reproductive structures, taxonomic comments, and information about the ecology and conservation of *C.restingae* are provided. Furthermore, the distribution and introduction of *C.africana* in the Americas is discussed. A total of eight names of *Christiana* species or synonyms described in four other genera (*Berrya*, *Carpodiptera*, *Entelea*, and *Speirostyla*) are lectotypified.

## ﻿Introduction

*Christiana* DC. (Malvaceae, Brownlowioideae) is a small genus with six species distributed across the Americas, Africa, Madagascar, and French Polynesia (Kubitzki 1995; Bayer et al. 1999; Hernández-Gutiérrez and Magallón 2019; Colli-Silva et al. 2025). It is closely related to two other genera of the subfamily, *Berrya* Roxb. from south and southeast Asia and *Carpodiptera* Griseb. from Central America, the West Indies, and East Africa, from which it differs by having glabrous and variegated seeds (Bayer and Kubitzki 2003; Barbosa-Silva et al. 2021). *Christiana* is comprised of monoecious or dioecious trees that have reflexed stigmas, one ovule per locule, fruits that are follicles or capsules with rudimentary wings or wingless, shiny, and with crustaceous endocarp, and variegated or mottled seeds (Bayer and Kubitzki 2003).

Kubitzki (1995) provided a new concept about the genus, revisiting its taxonomy and nomenclature, and recognized five species. In Brazil, taxonomic searches in the “Tiliaceae” (i.e., Malvaceae, Brownlowioideae and Grewioideae) made in São Paulo state (southeastern region) recorded *Christianamacrodon* Toledo (Souza and Esteves 2002), and for the state of Pernambuco (northeastern region) found only *C.africana* DC. (Tschá et al. 2002). Both studies included morphological descriptions and limited data on geographic distributions. *Christiana* has its highest species diversity in Brazil, where three species now are found (Coutinho 2025). Barbosa-Silva et al. (2021) reported new collection records of *C.mennegae* (Jans.-Jac. & Westra) Kubitzki and provided molecular data, a revised geographic distribution, and identified useful new characters for its determination, including seed stomata and glandular trichomes on vegetative and reproductive organs.

The Atlantic Forest is a global hotspot and contains 371 species of Malvaceae, of which 175 or almost half are endemic (Mittermeier et al. 2005; Flora e Funga do Brasil 2024). Recent research has underscored the richness of this phytogeographic domain with the description of new species in the family (Carvalho-Sobrinho 2013; Carvalho-Sobrinho et al. 2024; Coutinho et al. 2022; de Macedo et al. 2018; Ferreira and Bovini 2020; Ferreira 2021; Costa et al. 2022). Until now, only two species of *Christiana* were recorded for the Atlantic Forest; *C.macrodon* Toledo is endemic to the state of São Paulo and occurs in regions that intersect with the Cerrado phytogeographic domain (Toledo 1952; Souza and Esteves 2002) while *C.africana* is found sporadically in various countries in northern South America and with no identifiable phytogeographic domain. The latter species also is the only species in the genus with documented human uses for both material and medicine (Diazgranados et al. 2020).

The aim of this study is to present a synopsis of *Christiana*, providing comments about taxonomy, nomenclature, and geographic distribution, as well as to describe a new species endemic to the Brazilian Atlantic Forest.

## ﻿Materials and methods

The nomenclatural revision was performed by analyzing protologues and physical [housed in GH, K, MAC, MO, P, U, US, W; acronyms according to Thiers (2025)] and virtual (BISH, BM, G, HBG, JE, NY, R, RB, SP, VEN) types in herbaria, with support of JSTOR Global Plants (https://plants.jstor.org/) or physical samples.

Morphological descriptions of the new species were based on examination of material in CVRD, MO, NY, RB, US, and VIES herbaria. Exsiccatae were analyzed with a stereomicroscope to ascertain general characters. Leaves, fruits, and seeds from the specimen Lima 6114 of the species described herein were metalized and analyzed under a Scanning Electron Microscope (SEM) to examine minute morphological characters. Morphological terminology follows Harris and Harris (2001) and Radford et al. (1974) for most characters and Theobald et al. (1979) for trichomes.

The conservation status of the new species was assessed according to IUCN (2012) criteria and analyzed with support of the GeoCAT tool (Bachman et al. 2011). The distribution map for South American species was made using QGIS software v. 3.32 (QGIS Development Team 2024) with geographic coordinates taken from herbarium labels when available.

## ﻿Results

### ﻿Taxonomic treatment

#### ﻿ Christiana

Taxon classificationPlantaeMalvalesMalvaceae

DC., Prodr. 1: 516. 1824.

9172E5BB-5E0F-5394-AB13-73A3981F2467

Christannia Walp., Repert. Bot. Syst. 1(2): 360. 1842, sphalm. pro Christiana DC.
Speirostyla Baker, J. Linn. Soc., Bot. 25(171): 298. 1889. Type. Christianaafricana DC. (as Speirostylatiliifolia Baker)
Asterophorum Sprague, Bull. Misc. Inform. 1908(6): 249. 1908. Type. Christianaeburnea (Sprague) Kubitzki (as Asterophorumeburneum Sprague)
Tahitia Burret, Notizbl. Bot. Gart. Berlin-Dahlem 9(88): 609. 1926. Type. Christianavescoana (Baill.) Kubitzki (as Tahitiavescoana Baill.)

##### Type.

*Christianaafricana* DC.

##### Distribution.

A genus of six species found in Mexico, Central and South America, Africa, Madagascar, and French Polynesia, with Brazil where there are four species, two of which are endemic as its main center of diversity. The genus is primarily found in rainforest ecosystems.

##### Eponymy.

Candolle named this genus in honor of Christen (Christian) Smith (1785–1816), who collected in the Canary and Cape Verde islands and in the Congo.

### ﻿Key to the species of *Christiana* DC. (Malvaceae, Brownlowioideae)

**Table:** 

1	Leaf blades with dentate margins	**2**
--	Leaf blades with entire margins	**3**
2	Leaf blades finely dentate on the margins, especially apically; fruits winged	***Christianavescoana***
--	Leaf blades coarsely dentate on the margins; fruits not winged	***Christianamacrodon***
3	Leaf blades broadly ovate, bases cordate; gynoecium and capsules apocarpous	***Christianaafricana***
--	Leaf blades narrowly elliptic, elliptic, lanceolate, or oblanceolate, bases cuneate, rounded, or subcordate; gynoecium and capsules syncarpous	**4**
4	Inflorescences short peduncled; capsules conspicuously winged	***Christianamennegae***
--	Inflorescences long peduncled; capsules inconspicuously winged or wingless	**5**
5	Inflorescences umbellate with flowers attached to peduncles; capsules depressed-globose, inconspicuously winged	***Christianarestingae***
--	Inflorescences fasciculate with flowers on second order axes; capsules subturbinate to turbinate, wingless	***Christianaeburnea***

#### ﻿1. Christiana africana

Taxon classificationPlantaeMalvalesMalvaceae

DC., Prodr. 1: 516. 1824.

EFFC549D-CED0-564F-A47F-85CD2FB69CFF

Figs 1A, B , 2 , 3A

Christiana cordifolia Hook. f., Niger Fl. 238. 1849. Type. Nigeria, Quorra, oppos[ite] Stirling, s.d. (fr), *T. Vogel 200* (lectotype, here designated: K [K000241734; digital image!]; isolectotypes: K [K000241733; digital image!], K [K000241735; digital image!]).
Carpodiptera schomburgkii Baill., Adansonia 10: 181. 1872. (“*Carpodiptera*? *Schomburgkii*”). Type. Guyana [“British Guiana”]. Sine loc., [1840] (fl), *R.H. Schomburgk 800* [= ser. I, 800] (lectotype, here designated: P [P02143016]!; isolectotypes: B† [= F neg. no. 9254], K [K000381143]!, K [K000381144]!, P [P02143017]!, U [U 0006904]!, U [U0006905]!, W [W 0002458]!).
Christiana madagascariensis Baill., Bull. Soc. Linn. Paris 1(68): 542. 1885 (“C[hristiana]? madagascariensis”). Type. Madagascar. Semberano [sic], Dec 1879 (fl), *J.M. Hildebrandt 3262* (lectotype, here designated: P [P00077799; digital image!]; isolectotypes: JE [JE00003688; digital image!], K [K000241730; digital image!], P [P00077800!], W [1889-0089776]!).
Speirostyla tiliifolia Baker, J. Linn. Soc., Bot. 25(171): 299, pl. 50. 1889 (“*tiliæfolia*”). Type. Madagascar. Sembirano [sic], Dec 1879 (fl), *J.M. Hildebrandt 3262* (first-step lectotype, designated by Capuron, 1963, pg. 93, second-step lectotype, here designated: K [K000241730; digital image!]; isolectotypes: JE [JE00003688; digital image!], P [P00077799; digital image!], P [P00077800; digital image!], W [1889-0089776]!).

##### Type.

Congo. Sine loc., s.d. (fr), *C. Smith s.n.* (lectotype designated by Mabberley in Capuron and Mabberley 1999, pg. 22): BM [BM000795022; digital image!]; isolectotypes: GH [GH00247483]!, K [K000241731; digital image], K [K000241732; digital image!], MO [MO-036957] [n.v.], P [P00368004; digital image!]).

##### Description.

Trees, 2–16 m tall. Leaves: petioles 6–12 cm long, leaf blades 33 × 20.5–21 cm, widely ovate, concolorous to discolorous, bases cordate, margins entire, apices acuminate, stellate trichomes above and below. Inflorescences long peduncled; flowers attached on second or third order axes. Capsules apocarpous, 1.2–1.3 cm long, depressed-globose, wings absent.

**Figure 1.**
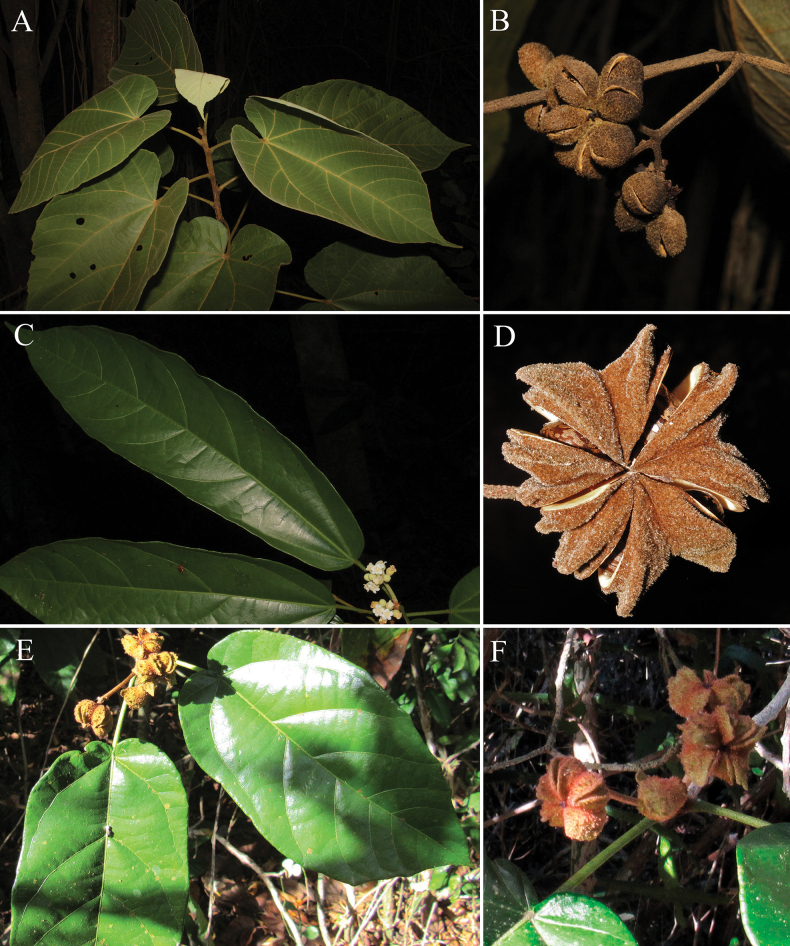
Several *Christiana* species found in Brazil **A***C.africana* leaves **B***C.africana* fruits **C***C.mennegae* leaves **D***C.mennegae* fruit **E***C.restingae* leaves **F***C.restingae* fruits. Photos: Rafael Gomes Barbosa-Silva (**A–D**), André Assis (**E–F**).

##### Distribution and habitat

**Figure 2.**
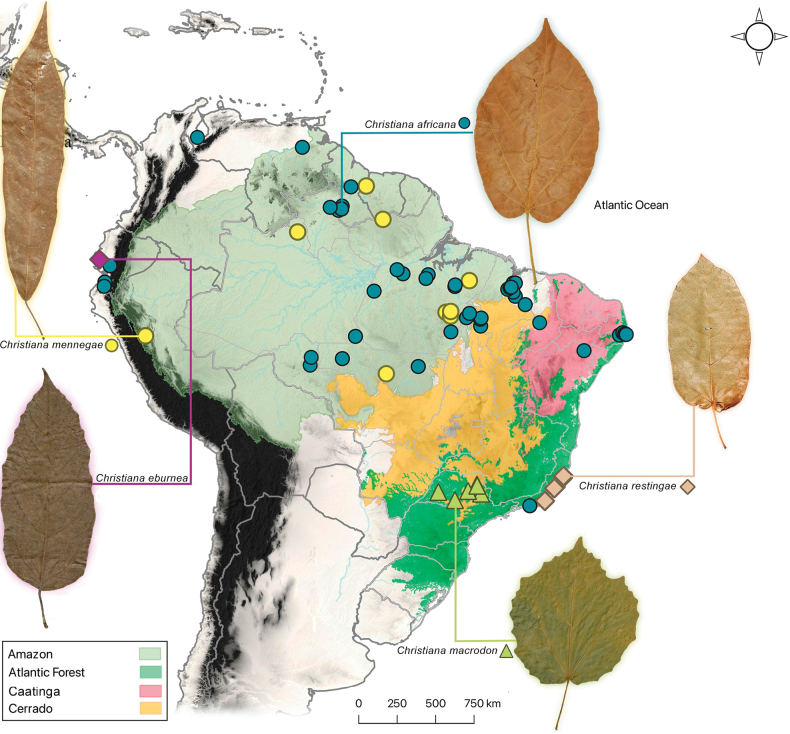
Geographic distribution of *Christiana* found in South America with leaf blade outlines for each species. Four phytogeographic domains (Amazonian Forest, Atlantic Forest , Caatinga, and Cerrado) are indicated for Brazil, the center of diversity for the genus.

(Fig. 2). Mexico, Belize, Costa Rica, Nicaragua, Venezuela, Guyana, Ecuador, and Brazil (Ceará, Maranhão, Mato Grosso, Pará, Pernambuco, Rio de Janeiro, Rondônia, Roraima and Tocantins), Central and West Africa, and Madagascar. In Brazil, Tschá et al. (2002) indicated the species occurred in Alagoas state but the specimen they cited (*M.T. Monteiro 22831*) is actually a species of Cordiaceae.

##### Phenology.

Flowering and fruiting specimens collected throughout the year.

##### Conservation status.

A widespread species, *Christianaafricana* can be assessed as Least Concern (LC) according to IUCN (2012) criteria.

##### Vernacular names.

Mexico: Patashtillo; Belize: Palo mulato; Nicaragua: Palo piedra; Costa Rica: Piedra; Venezuela: Cabeza de negro, however this common name is possibly misapplied as it usually refers to *Apeibatibourbou* Aubl. (Malvaceae, Grewioideae); Ecuador: Balsa real; and Brazil: Caaguassu, coração, fruta-de-pombo, gargaúba, and jangada.

##### Etymology.

The specific epithet refers to the African continent, where the type was collected.

##### Additional specimens examined.

**Mexico. Chiapas**: La Gloria, al E de Salto de Agua, 80 m, 17 Jun 1952 (fl), *F. Miranda 7536* (US). Mpio. Ococingo, a 3 km al S de Frontera Corozoza, sobre la orilla del río Usumacinata, 120 m, 2 Oct 1984 (fl), *E. Martínez S. 7983* (NY, 2 sheets). Mpio Ocosingo, al N de la Estación Chajul, camino a la Petatillera, 25 May 1999 (fr), *S. Sinaca C. 2624* (NY).

**Belize** [“British Honduras”]. Sine loc., 1926 (fl), *H.W. Winzerling III.7* (US). **Nicaragua. Río San Juan**: Rio San Juan, bosque situado al Oeste del Poblado de Boca de Sábalos (finca de Simeón Parrales Ulloa), 11°03'N, 084°31'W, 17 Jul 1994 (fl bud), *R. Rueda et al. 2097* (US!). **Without province**: “E. Nicaragua,” 1949 (st), *P.J. Shank 118* [= *Museum Yale School of Forestry Ser. No. 46904*] (WIS). **Costa Rica. Alajuela**: Canton de Los Chiles, R.N.V.S. Caño Negro, Llanuras de Los Guatusos, Caño Negro, 10°54'50"N, 084°46'05"W, 40 m, 17 Feb 1994 (fr), *J.F. Morales et al. 2386* (K, MO, NY). R.N.V.S. Caño Negro, Los Chiles. Caño Negro, camino a Playuelas, 10°50'10"N, 084°50'02"W, 40 m, 18 Feb 1994 (fr), *V. Ramírez et al. 261* (NY, US).

**Venezuela. Bolívar**: Parque Caroní, San Félix-Pto. Ordaz, Sep l965 (fl, fr), *L. Aristeguieta 5872* (NY, VEN, 3 sheets). Zulia: Dtto. Colón, alrededores de Casigua El Cubo, sector Los Manueles, vía Casigua-Palmira, cerca de la aldea Querrequerre, unos 12–15 km al N de Casigua, 31 Jul 1979 (im fr), *G.S. Bunting & M. Fucci 7749* (NY, VEN). **Guyana. U. Takutu-U. Essequibo Region**: 0–4 km N of Dadanawa, 100–120 m, 02°50'N, 059°31'W, 1 Jul 1989 (fl), *L.J. Gillespie 1949* (NY, US); Kanuku Mts., Rupununi R., Bush Mouth near Witaru Falls, 90–120 m, 03°04'N, 059°28'W, 11 Feb 1985 (fr). *M.J. Jansen-Jacobs et al. 154* (US); Rupununi Distr., Dadanawa, 120 m, 9 Jun 1995 (fl), *M.J. Jansen-Jacobs et al. 4000* (NY, U [digital image], US), ibid., 120 m, 10 Jun 1995 (fl), *M.J. Jansen-Jacobs et al. 4019* (NY, U [digital image], US); Siriuma [sic], Sep 1842 (fl), *M.R. Schomburgk 759* (A, B†); Roraima, 1842–43 (fl bud), *R.H. Schomburgk 474* (G, P, 2 sheets, W, 2 sheets). **Ecuador. Guayas**: Bosque protector Cerro Blanco, hacía la fabrica de cemento, 02°10'S, 079°58'W, 250 m, 18 Mar 2003 (fl), *X. Cornejo & C. Bonifaz 7615* (US). **El Oro**: Cerca a El Porvenir, 80 m, 15 Apr May 1979 (fr), *L. Albert de Escobar 1171* (QCA); Bosque Petrificado Puyango, al Norte Puyango, 03°52'S, 080°04'W, 450 m, 1 Jun 1995 (fr), *X. Cornejo et al. 4007* (US); Puyango and vicinity, 300–900 m, Aug 1978 (fr), *D.C. Daly 043* (NY). **Loja**: Bosque Petrificado Puyango, al Este Puyango, 03°52'S, 080°04'W, 300 m, 13 Apr 1996 (fl), *X. Cornejo & C. Bonifaz 5026* (US); Bosque Petrificado Puyango, camino a Querada Quemazones, 03°52'S, 080°04'W, 300 m, 14 Apr 1996 (fl), *X. Cornejo & C. Bonifaz 5054* (US). **Brazil. Ceará**: “M.N. Plantas da Comm. Scient. da Prov. Do Ceará,” s.d. (fr/st), *Fr. Allemão & M. de Cysneiros 136* (P, 2 sheets, R, 2 sheets). **Maranhão**: Fazenda São Francisco, estrada Alto Alegre-Lago Verde km 9, Município de Lago Verde, 21 Mar 1985 (st), *A.B. Anderson et al. 2026* (NY); Fazenda São Francisco, 11 km N of Km 337 of BR 316, 04°00'S, 044°56'W, 25 Sep 1980 (fr), *D.C. Daly et al. D260* (INPA [digital image], NY, US); Codó, 18 Jun 1907 (fl), *A. Ducke HG610* (INPA [digital image]); Sine loc., BR 135, Km 41–42, 18 Feb 1979 (fr), *A. Fernandes et al. s.n.* (EAC 5580 [digital image]); Maracassumé River Region, Campo do Cusins [sic, Caixas], 9 Sep 1932 (fr), *R. Froes 1874* (A, K, MICH, MO, NY, US, WAG [digital image], WIS); Beira do Rio Itacaiuna, Surubim, 19 Jun 1949 (fr), *L. Fróes & G.A. Black 24607* (NY, US); Estreito, ao lado da agrofloresta da fazenda Balneário rio das Pedras, 22 Feb 2005 (fr), *G. Pereira-Silva et al. 9585* (CEN [digital image]); Estreito, margen direita do Rio Tocantins/ foz do Rio Feio, 14 Jan 2008 (fl), *G. Pereira-Silva et al. 12610* (CEN [digital image]); Ilha dos Bótes, a duas léguas abaixo de Carolina, Rio Tocantins, 24 May 1950 (fr), *J.M. Pires & G.A. Black 1981* (IAN [n.v.], UB [n.v.], US); Engenho, município de Vitória do Arari, Campo temporariamente alagado, Cerrado, 30 Jun 1978 (fr), *N.A. Rosa 2484* (IAN [digital image], NY, U [digital image], UFG [n.v.]). Margem do Rio Grajaú, km 285 da Red. BR-316, Campo temporiamente alagado, 15 Dec 1978 (fr), *N.A. Rosa & H. Vilar 3003* (MG [n.v.], NY). **Mato Grosso**: Aripuanã, Salto dos Dardanelos, 10°09'S, 059°27'W, 11 Dec 1999 (fl), *B. Dubs 2639* (K, NY, UFMT); Source of the Jatuarana River, Machado River region, 17 Dec 1931 (fl), *B.A. Krukoff 1571* (A, K, MICH, NY, P, U [digital image]). **Pará**: São Geraldo do Araguaia, Santa Cruz do Araguaia, margem esquerda do rio Araguaia, 13 Jul 1995 (fr), *I. Aragão & M.N. Bastos 207* (IAN [digital image], MFS [digital image]); São Geraldo do Araguaia, Parque Estadual da Serra dos Martírios-Andorinhas, 29 Aug 2018 (fr), *L. Catarino & J.C. Freitas 2760* (IAN [digital image]); Rio Vermelho, região do Tocantins, 22 Apr 1951 (fl bud), *R.L. Fróes 26955* (IAN [digital image], NY, RB [digital image]); [Santarém], Rio Curuatinga, Planalto de Santarém, onde fei feito o levantamento estatístico florestal pelo IAN, SPVEA e FAO, 11 Mar 1955 (fl), *R.L. Fróes 31618* (IAN [digital image!], NY!); Beira do Rio Itacaiuna, Surubim, 19 Jun 1949 (fr), *R.L. Fróes & G.A. Black 24607* (IAN [digital image], US); Sine loc., Rio Araguaia, região de Xambioá, 11 Mar 1961 (fr), *E. Oliveira 1368* (IAN [digital image]); Rio São Manoel a 150 km da foz, limite Pará-Mato Grosso, várzea alta, 3 Jan 1952 (fl), *J.M. Pires 3765* (IAN [n.v.], US); Alto Tapajós, frSão Raimundo, foz do Rio Cururu, margem esquerda, 16 May 1977 (fr), *N.A. Rosa & M.R. Santos 1924* (K, INPA [digital image], NY); Tucuruí, Rio Tocantins, 03 Jun 1980 (fr), *M.G. Silva 5337* (INPA [digital image]); Itaituba, São Luiz, margem do Rio Tapajós, 07 Oct 1977 (fr), *M. Silva & L. Coêlho 2307* (INPA [digital image]); Breu Branco, Tucuruí, 3 Jun 1980 (fr), *M.G. Silva & C. Rosário 5337* (IAN [digital image!], NY!). **Pernambuco**: São Lourenço da Mata, Estação Ecológica do Tapacurá, 08°00'46"S, 034°57'01"W, 17 Aug 2001 (fr), *K. Almeida 183* (IPA [n.v.], CEN [digital image], NY); Recife, Mata de Dois Irmãos, 01 Jun 1950 (fl), *D. Andrade-Lima 488* (IAN); Recife, Dois Irmãos, Mata dos Macacos, 15 Mar 2013 (fl), *M.O. Barbosa et al. 1* (UFP [digital image]); Nazaré de Mata, 4 Jan 1959 (fl), *J. Coêlho de Moraes 2036* (A); Pernambuco, 1838 (fl), *G. Gardner s.n.* (K); [São Lourenço da Mata] Tapera, 15 Apr 1967 (fl), *A. Krapovickas 12871* (P); [São Lourenço da Mata] Tapera, border of the river Tapacurá, 10 Sep 1932 (fr), *B. Pickel 3098* (US); Recife, entrada da Guabiraba, margem da mata de Dois Irmãos, 14 Apr 1962 (fl), *S. Tavares 923* (HST [n.v.], UFP [digital image], US!); Camaragibe, Aldeia, 17 Feb 2013 (fl), *J.E.L. Torres & W.B. Santos s.n.* (HST 19975 [n.v.], HUEFS [000139628, digital image]). **Rio de Janeiro**: (Rio Janeiro) [illegible], s.d. (fr), *A. Glaziou 10314* (K, NY, P); Rio de Janeiro, São Cristóvão, 16 Apr 1883 (fl), *A. Glaziou 14513* (K, NY, P, R [digital image]; Rio Janeiro, Quinta, 12 Mar 1888 (st), *A. Glaziou 16705* (BR [digital image], K, LY [digital image], P); (Rio Janeiro), Quinta, 12 Mar 1888 (fl), *A. Glaziou 16709* (P); Vicinity of Rio de Janeiro & D’Ouro, *A. Glaziou s.n.* (P); Sine loc., s.d. (fr, st), *A. Glaziou s.n.* (NY p.p., P, US). **Rondônia**: Machadinho do Oeste, Tabajara, Rio Machado, 31 May 2015 (fr), *N.C. Bigio et al. 1633* (RON [digital image], US); Sine loc., território de Rondônia, 13 Aug 1975 (fr), *M.R. Cordeiro 541* (IAN [digital image]). **Roraima**: Boa Vista, ilha no meio do Rio Branco, ilha 1, parcela 2. Floresta de várzea; 15 Mar 2021 (fr); *R.G. Barbosa-Silva 1466* (MG); Territorio do Rio Branco, Rio Branco, Fazendas São Bento, Capela e Bom Intento, 3 Sep 1951 (fr), *G.A. Black 51-13316* (IAN [n.v.], P); Boa Vista, Rio Branco, 10 Sep 1943 (fr), *A. Ducke 1387* (A, NY, US, 2 sheets); Boa Vista (near Guyana), Rio Branco, Jul 1913 (fl), *J.G. Kuhlmann 3641* (U [digital image!]); Amazonas-Expedition, bei S. Marcos, Rio Branco, Jun 1909 (fl), *E. Ule 7871* (B†, K [K001214111; digital image], L [digital image], MG [n.v.]). **Tocantins**: Araguatins, margem do Rio Araguaia, 17 Apr 1976, (fl), *J.E. de Paula 989* (UB). **Without state or definite locality**: “Brazilian Amazon,” received 11 Jul 1933, *A. Ducke 189* [= *Museum Yale School of Forestry Ser. No. 23651*] (A); Altamira (PA)-Rio Xingu, Ilha Belo-Horizonte, 11 Oct 1986 (fr), *S.A. de M. Souza et al. 253* (IAN [n.v.], MO).

**Senegal. Tambacounda**: Berge Gambie, 11 Dec 1948 (fr), *J.-G. Adam 2465* (MO, P [digital image]); Nieri-ko, 26 Nov 1964 (fr), *J.-G. Adam 20028* (MO). **Guinea-Bissau.** Porto de Canamine, 11°08'30"N, 015°02'55'E, 8 Nov 1995 (fr), *F. Malaisse & V. Claes 14838* (WAG [digital image]). **Guinea.** Forécariah Préfecture: Sikhourou, Moribaya, derrrièrre Kambilaya, a côté de la rivière Kitemou, 09°41'26.3"N, 012°51'39.4"W [sic], 215 m, 7 Dec 2022 (fr), *G. Konomou et al. 1040* (K [digital image]). **Mali. Kayes**: Chutes des Félou près Kayes, 28 Nov 1958 (fr), *P. Jaeger 5666* (P [digital image], WAG [digital image]). **Sierra Leone. Kabala**: Kruto, 6 Feb 1966 (st), *J.-G. Adam 23590* (MO). **Liberia. Nimba**: Certos River bridge, E of Tapeta, 8 Oct 1961 (fr), *A.G. Voorhoeve 520* (WAG, 2 sheets [digital images]). **Côte d’Ivoire.** 47 km S of Bavé, 09°19'N, 004°10'W [sic], 20 Jun 1968 (fl), *J. Bokdam* 2868 (K [n.v.], MO, WAG [digital image]); Lamto Station, riverine forest of Bandama River, 06°15'N, 005°03'W, 12 Jul 1968, *F.J. Breteler 5280* (K [n.v.], US, WAG [digital image]). **Ghana.** Kwahu Tafo-Asuboni Rd., 24 Feb 1962 (fr), *J. Deaw Sp 650* (MO, US). Ejura, Jun 1930 (fl), *C. Vigne 2030* (A, MO, US). **Togo. Plateaux Region**: Badou, sur la route Badou-Danyi konta, Nov 1986 (fr), *K. Akpagana 1155* (TOGO [digital image]). **Nigeria.** Ibadan, Hadan, Oyo Aroba Hills F.R., Line 40, 18 Oct 1943 (fr), *A.P.D. Jones F.H.I. 4061* (K, MO). **Cameroon.** 1 km S of Badékok, W of km 45 of road, Yokadouma-Moloundou, left bank Badékok R., near conjunction with Boumba R., 475 m, 14 Jul 1965 (fl), *A.J.M. Leeuwenberg 6108* (BR [digital image], MO, WAG [digital image]); Likombe-Pflanzung, 15–35 km NE von Victoria; 50–100 m, Regenwald, Dec 1928 (st), *J. Mildbraed 10748* (A). **Central African Republic.** Région de Yalinga, Haut Oubangui, 1923–34 (fl), *G. Le Testu 3887* (BR, 2 sheets [digital images], MO, P [digital image]). **Republic of the Congo.** Modzaka (Oubangui), May 1889 (fl bud), *M. Thollon 63* (A, BR [digital image], MO, P [digital image]). **Democratic Republic of the Congo. Mai-Ndombe**: Bolobo (terr. Mushi), 25 Aug 1953 (fr), *G. Gilbert 14716* (BR [digital image], NY, US). **South Sudan** [“Anglo Egyptian Sudan”]. **Equatoria**: River Sue, 40 mi NE of Yambio, 24 Aug 1938 (im fr), *J.G. Myers 9376* (A). Tanzania [“Tanganyika”]. Utete Road, mile 10, 13 Jan 1940 (fl), *J.H. Vaughan 2939* (BR [digital image]). **Angola. Uige**: Damba (Congo), Feb 1942 (fl), *J. Gossweiler 13363* (LISC [digital image], WAG [digital image]). **Madagascar. Antsiranana**: Diana Region, Diana, Ambanja, Bemanevika, Bandrakorony, près d’une rivière affluente de la grande rivière de Bandrakorony sur la Péninsule d’Ampasindava, 13°45'45"S, 047°59'09"E, 47 m, 29 Jan 2000 (fl), *C. Rakotovao et al. 4316* (TAN, US). **Mahajanga**: Près du village d’Analanambe, campement 1, direction W à 2 km, Anjiamangirana I, Antsohihy, 15°09'21"S, 047°44'09"E, 150 m, 26 May 2000 (fr), *P. Ranaivojaona et al. 323* (P [n.v.], US). **Without province**: “Chiefly from North-west Madagascar,” recd. Sep 1887 (fl), *R. Baron 5742* (syntype of *Speirostylatiliifolia*: K, 2 sheets [K000241728, K000241729]).

##### Discussion.

*Christianaafricana* is easily distinguished from its congeners as it is the only species with a fully apocarpous gynoecium and capsule.

The African-American distribution of *Christianaafricana* is an unusual biogeographic pattern. Burret (1926) postulated a natural migration of *Christiana* from Madagascar westward to Africa and eastward via the Pacific to South America but there are no obvious adaptations for such long-distance dispersal and the American collections of *C.africana* do not support an indigenous presence; *C.africana* occurs sporadically and is invariably found in either secondary vegetation or near households. Kubitzki (1995), also confronted by the odd pantropical distribution of *Christiana*, simply reiterated Burret’s argument. There are relatively few examples of flowering plant taxa with an amphi-Atlantic disjunction (Thorne 1973; Renner 2004), which mostly can be attributed to long-distance dispersal. Renner (2004) suggested that unlike family or genus level disjunctions between America and Africa that fit this long-distance dispersal hypothesis, species level disjunctions often might be anthropogenic.

**Figure 3.**
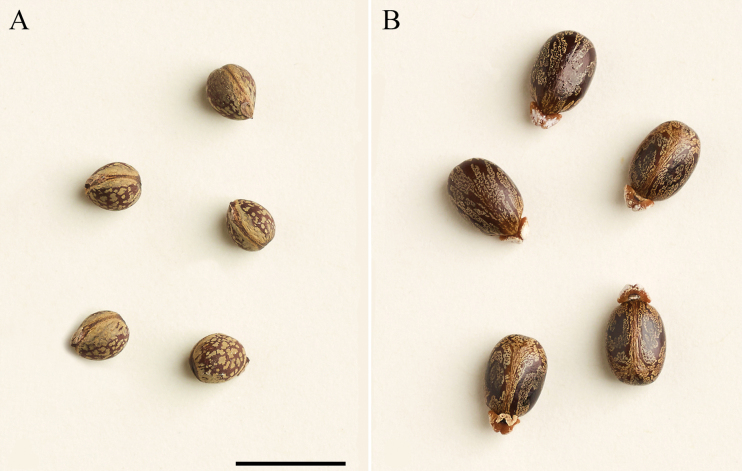
Morphological comparison of *Christiana* DC. and *Ricinus* L. seed **A***C.africana* DC. (Malvaceae) **B***R.communis* L. (Euphorbiaceae). (Vouchers: *C.africana*, *Pires & Black 1981*, US; *R.communis*, *Smith & Klein 10502*, US). Scale bar: 1 cm (**A, B**).

An alternative hypothesis is that *Christianaafricana*, at least, was introduced into the Americas by African slaves during the trans-Atlantic slave trade. Superficially, its seeds resemble in shape, color, and lack of indumentum those of castor bean (*mamona*), *Ricinuscommunis* L. (Euphorbiaceae) (Fig. 3), which is native to Africa, and which was brought to the Americas by slaves as a medicinal plant (see e.g., Voeks 1997, 2013; Carney 2013). This would explain its atypical distribution in the Americas and the hypothesis could be tested by a detailed genomic analysis of African and American populations. The four other South American species of *Christiana* are clearly native and although collected infrequently, they are found in undisturbed habitats.

*Christianaafricana* is “pollinated” (i.e., visited) by lepidoptera (fide *Cornejo & Bonifaz 5054*, in sched.).

Hooker (1849) based *Christianacordifolia* Hook. f. on a Vogel collection housed in Kew. In this herbarium, there are three duplicates of *Vogel 200*, and we designate here one of them (K000241734) as the lectotype of this name as it has Vogel’s detailed annotations about locality according to protologue.

*Carpodipteraschomburgkii* Baill. was described by Baillon (1872) based on a collection by *R.H. Schomburgk* (*800*) from Guyana. The material studied by Baillon is deposited in Paris (Stafleu and Cowan 1976) where there are now two duplicates of this collection. We designate here the most complete specimen with flowering branch (P02143016) as lectotype. An isolectotype at P appears to have Baillon’s notes and sketches while an isolectotype at B† was annotated by Burret.

Baillon (1885) described *Christianamadagascariensis* Baill. based on a collection by *Hildebrandt 3262* also housed in Paris. There are two duplicates of this collection now in this herbarium, and we designate one of them (P barcode P00077799) as the lectotype as it not only is representative but also has Baillon’s detailed annotations about the species.

*Speirostyla* Baker was proposed by Baker (1889) as a new monotypic genus endemic to Madagascar. Simultaneously, he described *S.tiliifolia* Baker based on two syntypes: *Baron 5742* and *Hildebrandt 3262*. Capuron (1963) distinguished between the syntype collections when he stated that the latter was the “Typus” of this name, but he failed to distinguish between duplicates of the Hildebrandt collection. A second-step lectotype therefore is designated here.

#### ﻿2. Christiana eburnea

Taxon classificationPlantaeMalvalesMalvaceae

(Sprague) Kubitzki, Bot. Jahrb. Syst. 116(4): 541. 1995.

D05F4FB2-2186-5EA2-A157-CE6DB5ACFBBF

Fig. 2

Asterophorum eburneum Sprague, Bull. Misc. Inform. Kew 1908(6): 249. 1908. Type. Ecuador. Guayas: Chonona [“Chonana”] near Guayaquil, Dec 1861 (fl), *R. Spruce 6260 bis* (lectotype, here designated: K [K000381141]!; isolectotype: K [K000381140]!).

##### Type.

Based on *Asterophorumeburneum* Sprague.

##### Description.

Trees, 4–5 m tall. Leaves: petioles 1.8–3.7 cm long, leaf blades 15.5–40.5 × 7.5–18 cm, ovate, discolorous, bases truncate to subcordate, margins entire, apices acuminate, stellate trichomes above and below. Inflorescences long peduncled; flowers on second order axes. Capsules syncarpous, 2–2.5 cm long, subturbinate to turbinate, wings inconspicuous.

##### Distribution and habitat

(Fig. 2). Endemic to Ecuador at 590–610 m elevation. *Christianaeburnea* occurs in areas of mangrove with influence of Savanna and Deciduous Forest. The report (Dorr 1999) of *C.eburnea* occurring in Peru was based on the misidentification of a specimen (*Schunke V. 5516*) properly referred to *C.mennegae* (Jans.-Jac. & Westra) Kubitzki as reported by Barbosa-Silva et al. (2021).

##### Phenology.

Flowering specimens collected in December and fruiting in August.

##### Conservation status.

*Christianaeburnea* is known from two localities, with only one specimen found within a protected area (Reserva Ecológica Manglares Churute). With an AOO of 8 km^2^, this species is assessed here as Critically Endangered (CR), under B2ab (ii, iv, v) criteria, according to IUCN (2012).

##### Vernacular names.

Unknown.

##### Etymology.

From the Latin ‘*eburneus*, *eborinus*’ meaning ivory-white with yellow tinge, a reference to the petal color according to the protologue.

##### Additional specimens examined.

**Ecuador. Guayas**: Naranjal, Reserva Ecologica Manglares – Churute, cerca a la Cumbre del Pancho Diablo de frente al Puerto de Gallo, 590––610 m, 02°25'S, 079°35'W, 13 Aug 1992 (fr), *C.E. Cerón 20141* (US [00976599]); Ad fluvium Daule prope Guayaquil, s.d. (fl, fr), *R. Spruce 6260* (BM [BM000554440; digital image], G [G00356894], MPU [MPU015290; digital image], P [P02143013], W [1889-0106457]); Guayaquil, s.d. (fr), *R. Spruce 6260* (P [P02143014]).

##### Discussion.

*Christianaeburnea* is recognized by its fasciculate inflorescences with flowers arranged on second order axes and subturbinate to turbinate capsules, features that differentiate it from *C.restingae*.

This species was described as a novelty discovered in the Kew Herbarium (K) and its protologue states “Ecuador. Chonana [sic] near Guayaquil, *Spruce 6260*.” A specimen in Herbarium Hookerianum (K) labeled by Sprague “*Asterophorumeburneum*, Sprague nov. gen et sp. Brownlowiearum” has Sprague’s notes and sketches and the exact locality cited in the protologue but it is numbered “6260 bis.” This specimen most closely matches the information given in the protologue and is designated here as the lectotype. We imagine that Sprague either miscopied the collection number or the word “bis” was added to the label later. Another specimen in Herbarium Hookerianum (K), also labeled by Sprague, has a copied label with the locality “prope Guayaquil” and the number 6260 bis. It clearly is a duplicate of the former.

Additional Spruce material (BM, G, MPU, and P) labeled “Ad fluvium Daule prope Guayaquil” has the number “6260” as cited in the protologue but this material lacks the precise locality that was given even though Chonona, an indigenous locality, eventually was subsumed by the municipality of Daule (Jijón y Camaño 1919). Given the locality conflict, these specimens are considered original but not type material. Finally, two herbarium sheets of *Spruce 6260* in Paris (P02143014 and P02143015) lack original labels yet have Baillon’s sketches and notes; the latter sheet consisting of notes only.

#### ﻿3. Christiana macrodon

Taxon classificationPlantaeMalvalesMalvaceae

Toledo, Arq. Bot. Estad. São Paulo, n.s., f.m., 3: 29, pl. 8. 1952 [“1945”].

BE060D66-3F61-5ED4-A269-A0160DB0B6FB

Fig. 2

##### Type.

Brazil. São Paulo: Jardinópolis, mata à beira do Rio Pardo, 19 Nov 1947 (fl bud, fr), *M. Kuhlmann 2009* (lectotype, here designated: SP [SP001662; digital image!]; isolectotypes: HBG [HBG-512735; digital image!], HBG [HBG-512736; digital image!], MAC [MAC0055147]!, NY [01795748; digital image!], RB [RB 553263; digital image!], SP [SP001663; digital image!]).

##### Description.

Shrubs to trees, 1.5–3 m tall. Leaves: petioles 5–7 cm long, leaf blades 10–15 × 8–12 cm, widely ovate to circular, concolorous, bases cordate, margins coarsely dentate, apices acuminate, stellate trichomes above and below. Inflorescences short peduncled; flowers attached to peduncle. Capsules syncarpous, 1–1.2 cm long, transversely ellipsoid, wings absent.

##### Distribution and habitat

(Fig. 2). Endemic to Brazil, where it is found only in São Paulo state. This species occurs in Savanna and semi-deciduous seasonal forest (Atlantic Rainforest) at 530–600 m elevation.

##### Phenology.

Flowering specimens collected in November, and fruiting in April and September.

##### Conservation status.

Endangered (EN) (Fernandes and Amorim 2018).

##### Vernacular name.

Algodoeiro.

##### Etymology.

From the Greek ‘*macr*, *macro*’ meaning ‘long, large, great’, and ‘*odus*, *odon*, *odontos*’ meaning ‘teeth’, the specific ephitet probably referring to the conspicuously dentate leaf blade margins.

##### Additional specimens examined.

**Brazil. São Paulo**: Adamantina, Estação Experimental do IAC, 05 Sep 1995 (fr), *L.C. Bernacci et al. 1968* (SPF [digital image]); Porto-Ferreira Reserv. Estad. Porto Ferreira, 17 Dec 1980 (fl bud), *J.E.A. Bertoni 16889* (RB [RB 312587; digital image]); Gália, 15 Jun 2005 (st), *M.R. Gorenstein 22202* (ESA [digital image]); Ribeirão Preto, margem do Córrego Labareda, 11 Nov 2001 (fl bud), *O. Kotchetkoff-Henriques & A. Furlan 623* (ESA [digital image], IAC [digital image], SPFR [digital image]); Jardinópolis, margem do Rio Pardo, s.d. (st), *M. Kuhlmann 2965* [= Herb. No. 50524] (HAS [digital image], IAN [n.v.], SP [n.v.], US [00627713]); Matão, Fazenda Cambuhy, 14 Apr 1994 (fr), *V.C. Souza et al. 5650* (SP [n.v.], SPF [digital image], UNIP [digital image]).

##### Discussion.

Both *Christianamacrodon* and *C.vescoana* have dentate leaf blades, but the former species is distinguished by its coarsely dentate margins and fruits not winged (vs. finely dentate margins and fruits winged in the latter).

The protologue states that the “Typus” is a collection (N.° 43.987) in the herbarium at São Paulo (SP) but inasmuch as there now are two sheets with this number in that herbarium, one is designated here as the lectotype (SP barcode SP001662), as it has fruit.

#### ﻿4. Christiana mennegae

Taxon classificationPlantaeMalvalesMalvaceae

(Jans.-Jac. & Westra) Kubitzki, Bot. Jahrb. Syst. 116(4): 541. 1995.

0BCED60D-73C0-5474-A425-B958422DE2D2

Figs 1C, D , 2

Asterophorum mennegae Jans.-Jac. & Westra, Proc. Kon. Nederl. Akad. Wetensch., C, 86(3): 377, figs. 1–4. 1983. Type. Suriname: [Sipaliwini], ‘‘Morro Grande’’ camp-forest island, 6 km W of ‘‘Morro Grande’’ dome, Sipaliwini savanna area on the Brazilian frontier, 04 Nov 1968 (lf, fl, fr), *F.H.F. Oldenburger, R. Norde, & J.P. Schulz ON415* (lectotype, designated by Barbosa-Silva et al. 2021, pg. 4, 18): U [U0006902; digital image!], isolectotypes: K [K000381142]!, MO [MO-036999]!, NY [00415374; digital image!], P [P02143012]!, U [U0006903; digital image!], VEN [VEN409940; digital image!]).

##### Type.

Based on *Asterophorummennegae* Jans.-Jac. & Westra.

##### Description.

Trees, 5–17 m tall. Leaves: petioles 1.8–5.5 cm long, leaf blades 12.3–29.5 × 5–9 cm, narrowly elliptic, lanceolate or oblanceolate, concolorous, bases rounded to obtuse, rarely subcordate, margins entire, apices acuminate to cuspidate, stellate-multiangulate and glandular trichomes above and below. Inflorescences short peduncled; flowers attached to peduncle. Capsules syncarpous, 1.2–1.8 cm long, depressed-globose, wings conspicuous.

##### Distribution and habitat

(Fig. 2). Found in Suriname, Peru, and Brazil (Amazonas, Mato Grosso, and Pará states) at 500–600 m elevation. This species grows in Amazonian Rainforest and areas of evergreen seasonal forest in Savanna (Barbosa-Silva et al. 2021).

##### Phenology.

Flowering specimens collected in January, September, and November and fruiting in January, April, July, September, and November.

##### Conservation status.

Least Concern (LC) (Barbosa-Silva et al. 2021).

##### Vernacular name.

Brazil: Tartaruguinha.

##### Etymology.

The specific epithet honors Dr. Alberta M.W. Mennega, plant taxonomist and wood anatomist (Jansen-Jacobs and Westra 1983).

##### Additional specimens examined.

**Suriname.** Area of Kabalebo Dam project, distr. Nickerie, 4o-5o NB, 57°30’-58°WL, 30–130 m, forest along trail to Wonotobo about ½ km W of road km 109, 24 Sep 1980 (st), *J.C. Lindeman et al. 589* [= Woodsample Uw 26517] (NY). **Peru. San Martín**: Prov. Mariscal Cáceres; Tocache Nuevo, nor oeste del Caserío de Bambamarca, 500–600 m, 15 Nov 1972 (fr), *J. Schunke V. 5516* (F [F1780039; digital image], MO, NY, W). **Brazil. Amazonas**: margem de um igarapé que nasce na Serra de Aracá, 28 Jul 1977 (fr), *N.A. Rosa & M.R. Cordeiro 1699* (IAN, 2 sheets [digital images], RB [digital image]). **Mato Grosso**: Itaúba, Resgate de Flora da UHE Colíder, lote G de supressão, 28 May 2015 (fr), *J.P. Battisti s.n.* (CNMT [7492; digital image], MBM [403702; digital image]). **Pará**: Parque Zoobotânico, 05 Nov 2019 (fl), *R.G. Barbosa-Silva et al. 1424* (MG); Paragominas, ramal principal após entrada para sede, 29 Jun 2023 (fr), *E.D. Cruz et al. 1629* (IAN [digital image]); Nova-Canaã dos Carajás [Canaã dos Carajás], 06 Jan 2001 (fl, fr), *L.C.B. Lobato 2624* (MG); Km 141 da rodovia Belém-Brasília, 19 Fev 1960 (st), *E. Oliveira 538* (IAN [digital image]); Km 167–173 da estrada Belém-Brasília, 25 Apr 1960 (fr), *E. Oliveira 565* (IAN [digital image]); Sine loc., mata da Cia. Pirelli, Fazenda Uriboca, Jul 1958 (fr), *J.M. Pires 7041* (IAN, 2 sheets [digital images]); Marabá, [Mun. Parauapebas], Serra dos Carajás, 29 Nov 1988 (fr), *N.A. Rosa & F.C. Nascimento 5084* (K, MG [n.v.]); Parauapebas, Floresta Nacional de Carajás, imediações do Parque Zoobotânico, 06 Sep 2018 (fl, fr), *D.C. Zappi et al. 4562* (MG).

##### Discussion.

*Christianamennegae* is recognized by its short-peduncled inflorescences.

For additional details see the extensive and detailed discussion in Barbosa-Silva et al. (2021).

#### ﻿5. Christiana restingae

Taxon classificationPlantaeMalvalesMalvaceae

T.S.Cout., Barb.Silva & Dorr sp. nov.

87549214-CD5C-583D-BA32-07D2D53F1227

urn:lsid:ipni.org:names:77357319-1

**Figure 4.**
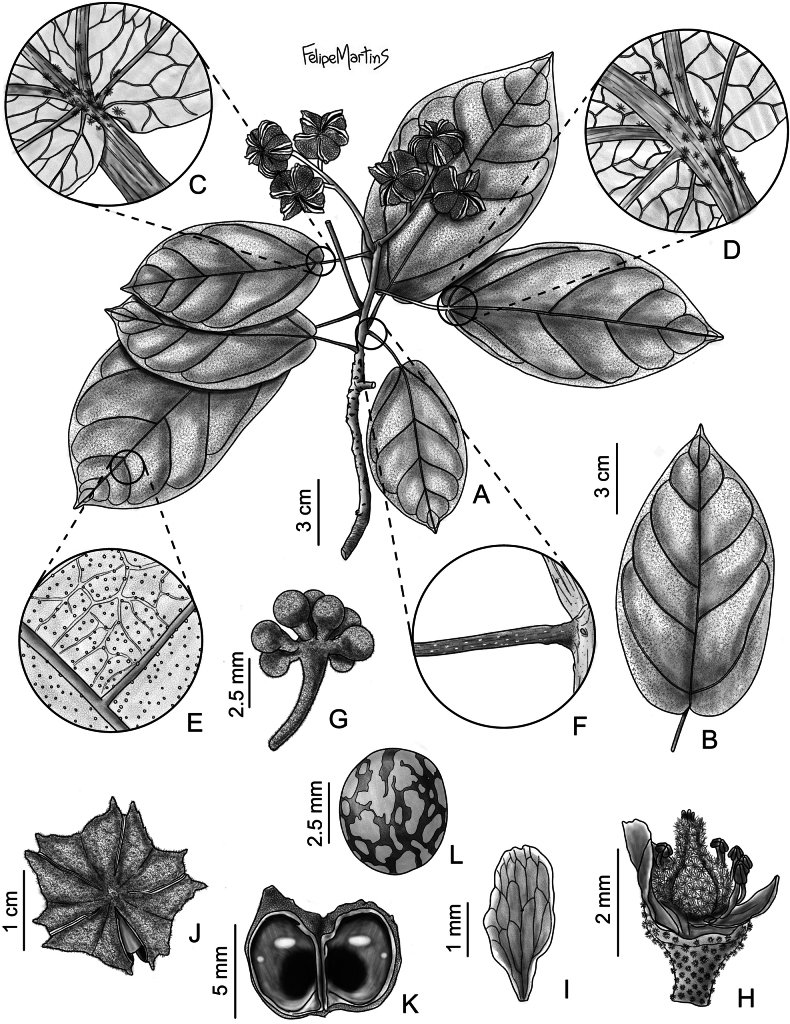
*Christianarestingae* T.S. Cout., Barb.Silva & Dorr **A** habit **B** leaf **C** detail of the base of the adaxial surface of the leaf blade showing concentration of stellate-multiangulate trichomes **D** detail of the base of the abaxial surface of the leaf blade showing concentration of stellate-multiangulate trichomes **E** detail of abaxial surface of the leaf blade showing glandular sessile trichomes **F** petiole **G** inflorescence with flower buds **H** gynoecium and part of androecium **I** petal **J** capsule **K** capsule opened showing shiny endocarp **L** seed.

**Figure 5.**
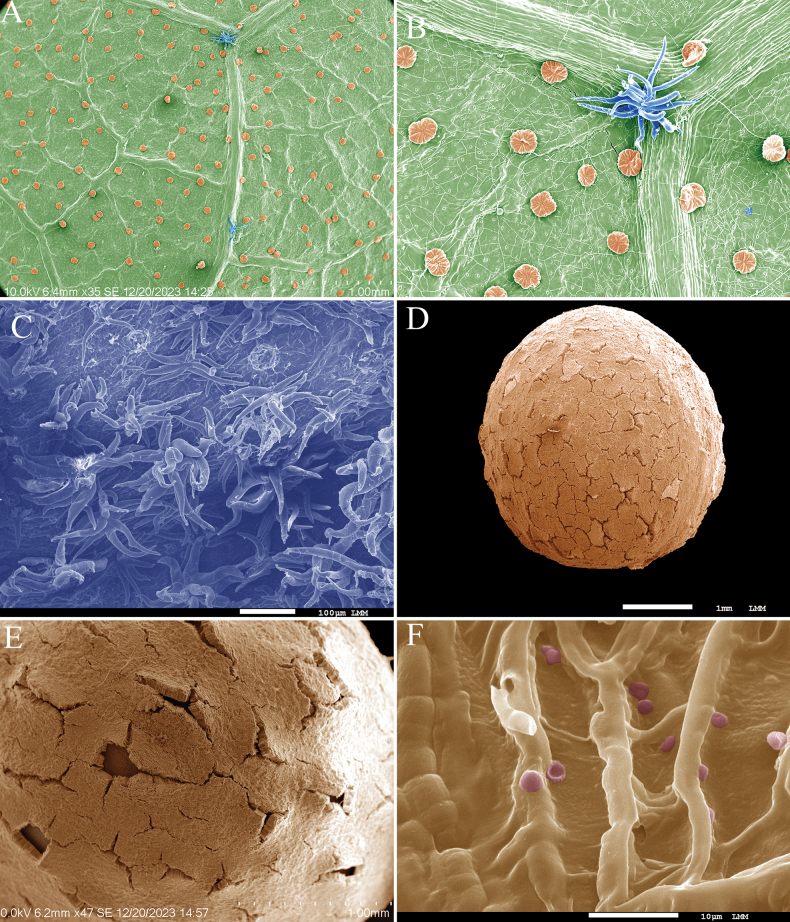
SEM images of *Christianarestingae* T.S. Cout., Barb.Silva & Dorr **A** abaxial leaf surface showing glandular trichomes (orange) and stellate-multiangulate trichomes (blue) **B** detail of the abaxial leaf surface **C** epicarp **D** seed **E** seed surface displaying the openings and fissures characteristic of this species **F** detail of scattered micropapillae on the seed surface.

Figs 1E, F , 2 , 4 , 5

##### Type.

Brazil. Espírito Santo: Vila Velha, Interlagos, 20 Jun 1996 (fr), *O. Zambom & M. Fernandes 286* (holotype: VIES [VIES010759]!).

##### Diagnosis.

*Christianarestingae* resembles *C.mennegae* but can be distinguished by its elliptic (vs. narrowly elliptic, lanceolate, or oblanceolate) leaf blades, calyces with stellate only (vs. stellate and glandular) trichomes, and capsules with inconspicuous (vs. conspicuous) wings.

##### Description.

Trees, 4–11 m tall, functionally dioecious. Branches whitish, cylindrical, rugose, lenticels circular to oblong, 0.5–1 mm long, old branches glabrescent, trichomes stellate-multiradiate, sessile, ferrugineous, sparsely distributed, stipules caducous. Leaves alternate, spirally arranged; petioles 1.3–3 cm long, discolorous when compared to the branches, vinaceous, inconspicuously striate, not canaliculate, terete, not decurrent, sparsely pilose, trichomes stellate-multiradiate, ferrugineous, more concentrated near the branches; leaf blades entire, coriaceous, nitid when dry, concolorous to quickly discolorous, 5.5–13.5 × 3–7 cm, elliptic, bases round to subcordate, margins plane, entire, apices acuminate to cuspidate, glabrous or glabrescent above and below, but with stellate-multiradiate trichomes sparsely distributed along the veins, and glandular-sessile trichomes on the blade below; venation actinodromous, 6–8 pairs of secondary veins, 2 pairs basal, impressed above, prominent below. Inflorescences axillary, umbellate with flowers attached to peduncles; bracts caducous; peduncles whitish, striate, 5–5.5 cm long, glabrous to glabrescent, trichomes sparse. Floral buds globose, ca. 2.5 × 2.5 mm. Flowers functionally unisexual; bracteoles c. 3.5 × 0.9 mm, elliptic, abaxial surface pubescent; pedicels 1.8–2 mm long, densely pubescent, trichomes stellate-multiradiate, ferrugineous, sessile. **Pistillate flowers**: calyx gamosepalous, 5-merous, cupuliform, c. 4.5 mm long, with apical lobes free, pubescent abaxially, trichomes stellate-multiradiate, lobes c. 2.5 × 2 mm, ovate, apex acute. Corolla dialypetalous, 5-merous, white, petals c. 2.8 × 1.3 mm, obovate, glabrous, apices rounded. Androecium polystemonous, staminodes 1.4–2 mm long, filaments 1.2–1.8 mm long, glabrous, anthers c. 0.3 mm long, divergent. Gynoecium 5-carpellate, ovary ca. 1.2 × 1 mm, depressed globose, densely pubescent, trichomes stellate-multiradiate, style hirsute, trichomes stellate-multiradiate, stigma not observed. **Staminate flowers**: not observed. Fruit a capsule, woody, syncarpous, c. 1.5 cm long, 1.3–1.7 cm diam., depressed-globose, 4–5-locular, inconspicuously winged, c. 0.6 × 0.6 mm, densely pubescent abaxially, glabrous, lustrous internally; seeds 1 per locule, 5 × 4 mm, ellipsoid, variegated, gray with brownish marks, glabrous.

##### Distribution and habitat

(Fig. 2). *Christianarestingae* is known only from Espírito Santo state, in Vila Velha and Presidente Kennedy municipalities, and in the northeast of Rio de Janeiro state. This is the only species of the genus occurring in the state of Espírito Santo. The new species is found in the Atlantic Forest phytogeographic domain, where it grows in Dense Ombrophylous Forest, as well as in Restinga, at 5–300 m elevation.

The Brazilian coast has an intense history of plant collecting, especially in the coastal region of the states of Espírito Santo and Rio de Janeiro. Recently, Dutra et al. (2022) with the Espírito Santo Flora Project strengthened floristic and taxonomic studies in the state, including new collections in Restinga areas (Guarnier et al. 2022).

##### Phenology.

Flowering specimens collected in January and fruiting in January, May, June, and August.

##### Conservation status.

*Christianarestingae* could be assessed as Endangered (EN) because its EOO is 1,230 km^2^ (< 5,000 km^2^) or Critically Endangered (CR) because its AOO is 8 km^2^ (< 10 km^2^). The IUCN (2012) recommendation is that the taxon be classified under the highest threat category. We therefore consider the species Critically Endangered (CR) under B2ab (i, ii, iii) criteria. In addition, *C.restingae* grows in Atlantic Forest and Restinga, vegetation types with an intense history of deforestation and human occupation.

##### Vernacular name.

Guaxumbão.

##### Etymology.

The specific epithet refers to Restinga, vegetation typical of the Brazilian coast, characterized by sandy soil, where the new species is usually found.

##### Paratypes.

**Brazil. Espírito Santo**: Presidente Kennedy, 21°16'07"S, 040°57'58"W, 5 m elev., 12 Aug 2020 (fr), *A.M. Assis & R.S. Cribari 4899* (VIES). Vila Velha, Convento da Penha, 300 m, 11 May 2007 (fr), *D.A. Folli 5755* (CVDR [digital image], US [01317134]); ibid., 300 m, 11 Sep 2009 (fr), *D.A. Folli 6409* (CDRV [digital image], US [01317133]); Vila Velha, Interlagos, 22 May 1996 (fr), *O. Zambom & M. Fernandes 283* (VIES), ibid., 31 Jan 1996 (fl, im fr), *O. Zambom et al. 222* (VIES). **Rio de Janeiro**: Armação de Búzios, 31 Aug 2003 (fr), *H.C. Lima et al. 6114* (MBM [digital image], NY [03987289], RB [digital image]); São Francisco de Itabapoana, Estação Ecológica de Guaxindiba, 17 Jul 2018 (fr), *H.C. Lima et al. 8683* (RB, 2 sheets [digital images]); Rio das Ostras, Jul 2004 (fr), *A. Oliveira & D. Oliveira 1016* (RB).

##### SEM.

The abaxial surface of the leaves has many sparse glandular trichomes and adjacent to the veins near the base it has stellate-multiangulate trichomes (Fig. 5A, B). The abaxial surface of the epidermis has cells shaped like irregular polygons (Fig. 5B). The epicarp has stellate multiangulate trichomes (Fig. 5C).

The seed has several openings and crevices (Fig. 5D, E) and its surface has small sparse papillae (Fig. 5F).

##### Discussion.

*Christianarestingae* is described here three decades after it was first collected by O. Zambom and M. Fernandes. Ironically, it is worth mentioning that most of the large botanical research centers in Brazil are concentrated in the central region of the Atlantic Forest, and this is where most Brazilian plant taxonomists are based. This underscores the lack of attention *Christiana* has received in recent years (Barbosa-Silva et al. 2021) and the paucity of specialists knowledgeable about certain groups of plants.

*Christiana* is a genus with homogeneous morphology, sharing stellate trichomes on the vegetative and reproductive parts including inflorescences and flowers. Using a Scanning Electron Microscope (SEM), Barbosa-Silva et al. (2021) discovered the occurrence of simple, two-armed, and glandular trichomes on the petioles and leaf blades of *C.mennegae*, characters known only from this species. Leaf shape and capsule morphology can be useful to distinguish the different species. *Christianaafricana*, the most widespread species of the genus, has an apocarpic gynoecium and fruit, which is an apomorphy. On the other hand, *C.macrodon* is the only species with leaf blades that are serrate along the entire length of their margins. Analyzing the type of *C.vescoana*, it was observed that in a few specimens the leaf blades near the apex also have this leaf margin characteristic, albeit less pronounced.

*Christianarestingae* is characterized especially by its elliptic leaf blades and fruits with inconspicuous wings. It has the shortest leaf length among the species of the genus, with mature leaf blades as small as 5.5 cm long. The new species differs from *C.africana* and *C.macrodon* by its elliptic leaf blade with rounded to subcordate (vs. widely cordate and cordate) bases. It also is distinguished from *C.africana* by its syncarpous (vs. apocarpous) capsules and from *C.macrodon* by leaf blades with entire (vs. dentate) margins. Although, morphologically, the new species is more similar to *C.mennegae* in having nitid leaves when dried, glabrescent, and with trichomes especially on the veins, *C.restingae* is distinguished by having smaller leaf blades (5.5–13.5 × 3–7.5 vs. 12.3–29 × 5–9 cm) that are elliptic (vs. narrowly elliptic, lanceolate or oblanceolate), and capsules 1.3–1.7 cm in diam. and with inconspicuous wings (vs. 3.8 cm in and conspicuous wings).

The analysis of structures in SEM brings new insights into understanding the genus, which exhibits various characteristics that can aid in species differentiation. For instance, the leaves of *Christianarestingae* possess more glandular trichomes than *C.mennegae* and have stellate-multiangulate trichomes, whereas *C.mennegae* only has stellate rotate trichomes on its leaves (Barbosa-Silva et al. 2021). Additionally, with SEM, the cell walls of the epidermis of *C.restingae* are visible, a character not seen in *C.mennegae*. The epicarp of *C.restingae* also exhibits a high concentration of stellate-multiangulate trichomes similar to *C.mennegae*. The seed of *C.restingae* also has maculae that are medium brown with darker irregular marks like other species in the genus; however, only *C.restingae* has these openings and crevices (Fig. 5E), which even can be observed in herbarium material without the aid of magnification. Although these openings and fissures have been found in all fruit-bearing material, we are not sure if they are found in fresh material or if this character occurs only during the drying process of specimens. The seed of *C.restingae* does not have stomata, a character that was found to be very interesting in *C.mennegae* and is also present in other Malvaceae species, although it is rare in Angiosperms (Paiva et al. 2006; Barbosa-Silva et al. 2021).

Regarding geographic distribution, *Christianarestingae* is endemic to Espírito Santo and Rio de Janeiro states, occurring in Atlantic Forest and Restinga, sharing the vegetation type with *C.africana* (Amazonia and Atlantic Forest) and *C.macrodon* (Atlantic Forest and Cerrado) (Coutinho 2025). *Christianamennegae* and *C.restingae* do not occur sympatrically since the first species occurs in Brazil only in the Amazonian Domain (Secco 2000; Barbosa-Silva et al. 2021). With the discovery of *Christianarestingae*, the number of species of the genus in Brazil reaches four, making the country the center of diversity. Of the six known species of the genus, two are endemic to Brazil and its Atlantic Forest.

It is worth noting that with the Flora do Brasil 2020 project (BFG 2021), various taxonomists were encouraged to monograph different groups. Consequently, plant groups that had long gone without taxonomic revisions, that were neglected, or that were known to be of little interest, were revised. Thus, numerous results have been published in recent years as a result of this careful process of revision in these groups (Giulietti 2020; Andrino et al. 2022; Antar et al. 2022; Asprino et al. 2024; Barbosa-Silva 2024). The same factor also has driven recent studies on the genus *Christiana* (Barbosa-Silva et al. 2021), as well as the data presented here.

#### ﻿6. Christiana vescoana

Taxon classificationPlantaeMalvalesMalvaceae

(Baill.) Kubitzki, Bot. Jahrb. Syst. 116(4): 541. 1995.

5258E624-DE99-5E4D-B25D-D3A608C8E6F4

Berrya vescoana Baill., Adansonia 10: 240. 1872. Type. Tahiti. Sine loc., 1847 (fr), *J.N.E. Vesco s.n.* (lectotype, here designated: P [P00637093; digital image!]; isolectotypes: BISH [BISH1003216; digital image!], P [P00637094; digital image!], P [P00637095; digital image!], P [P04756685; digital image!], P [P04756686; digital image!]).
Entelea tahitensis Nadeaud, Énum. Pl. Tahiti 69. 1873. Type. Tahiti, [Ravins de Papaihonu, 600 m.], s.d. (fr), *J. Nadeaud 439* (lectotype, here designated: P [P04756684; digital image!]; isolectotypes: BISH [BISH1003217; digital image!], G, 3 sheets [G00393886; digital images!], P [P04756683; digital image!], P [P00637089; digital image!], P [P00637090; digital image!], P [P00637091; digital image!], P [P00637092; digital image!]).
Berrya tahitensis (Nadeaud) Drake, Ill. Fl. Ins. Pacif.: 125. 1890 [“1886”] (“B. (?) Tahitensis”). Type. Based on Enteleatahitensis.
Tahitia vescoana (Baill.) Burret, Notizbl. Bot. Gart. Berlin-Dahlem 9: 607, 610. 1926. Type. Based on Berryavescoana.
Tahitia tahitensis (Nadeaud) S.L. Welsh, Fl. Societensis: 282. 1998. Type. Based on Enteleatahitensis.

##### Type.

Based on *Berryavescoana* Baill.

##### Description.

Trees, 5–8 m tall. Leaves: petioles 2.5–6.5 cm long, leaf blades 11–21 × 7.3–11 cm, widely ovate, concolorous to slightly discolorous, bases cordate, margins slightly dentate apically, apices acuminate, stellate trichomes above and below. Inflorescences short peduncled; flowers attached to peduncle. Capsules syncarpous, c. 1 cm long, depressed-globose, wings conspicuous.

##### Distribution and habitat.

Found only on the islands of Moorea and Tahiti in French Polynesia at 100–280 (–600) m elevation. The species is poorly known with relatively few recent collections.

##### Phenology.

Fruiting specimens collected in April, May, and June.

##### Conservation status.

*Christianavescoana* has a restricted and endemic population and presents an AOO of 20 km^2^ and EOO of 248.140 km^2^. It is assessed here as Critically Endangered (EN), under D1 criterion as it has a reduced number of mature individuals, according to IUCN (2012).

##### Vernacular names.

Unknown.

##### Etymology.

The specific epithet honors Jean Nicolas Eugène Vesco, Naval surgeon and botanist, who collected the type.

##### Additional specimens examined.

**French Polynesia. Moorea**: Vallée de Maharepa, 23 Apr 2004 (fr), *J.F. Butaud 439* (P, 2 sheets [P05253217, P05253221; digital images]); Mt Raaiu [sic], 16 Apr 1898 (fr), *J. Nadeaud s.n.* (P [P05253216; digital image]). **Tahiti**: Vallée du Panaruu, 21 May 1896 (fr), *J. Nadeaud s.n.* (P, 3 sheets [P05253218, P05253219, P05253220; digital images]); Sine loc., 1870 (fr), *M. Pancher s.n.* (P [P05253224; digital image]); Vallée de Faataua, 09 May 2005 (st), *W. Teamotuaitau 26* (P [P05253226; digital image]); Punaruu, 02 Jun 2005 (fr), *W. Teamotuaitau 35* (P [P05253222; digital image]). Sine loc., sine coll. (P [P05253225; digital image]); Sine loc., 1855 (fr), *M. Vieillard & M. Pancher s.n.* (P [P05253227; digital image]).

##### Discussion.

*Christianavescoana* shares dentate leaf margins with *C.macrodon*, but it is distinguished by characters previously mentioned (and also given in the key).

For additional details see Florence (2004).

The description of *Berryavescoana* was based on an unnumbered collection made by M. Vesco in Tahiti: this collection, including duplicates, is now housed in the herbarium in Paris (P). Kubitzki (1995) noted the type was in P, but only wrote “*Vesco a. 1847* (P)” where “1847” refers to the year of collection, not a collection number. Five duplicates of this unnumbered collection are found now in P, none of them have the “a” noted by Kubitzki, and consequently, we designate the most complete specimen with fruits and seeds (P barcode P00637093) here as the lectotype. It is also the same specimen that Florence (2004) considered to be the “holotype.”

When Nadeaud (1873) described *Enteleatahitensis* he associated it with a single collection (*Nadeaud 479*) for which he also indicated there were duplicates (“un nouvel examination de mes échantillons …”). There therefore is no holotype as indicated by Florence (2004) who stated that one was deposited in G. Additionally, Florence’s statement cannot be interpreted as designating a lectotype since it contravenes the rules of the ICN (Turland et al. 2018; Arts. 7.3, 9.23). Here, we designate as lectotype the only specimen in P (P barcode P04756684) with fruits.

## Supplementary Material

XML Treatment for Christiana

XML Treatment for Christiana africana

XML Treatment for Christiana eburnea

XML Treatment for Christiana macrodon

XML Treatment for Christiana mennegae

XML Treatment for Christiana restingae

XML Treatment for Christiana vescoana

## ﻿Additional information

### Author ORCIDs

Thales Silva Coutinho https://orcid.org/0000-0002-2173-4340

Rafael Gomes Barbosa-Silva https://orcid.org/0000-0002-0668-4840

Laurence J. Dorr https://orcid.org/0000-0001-7157-363X

### Data availability

All of the data that support the findings of this study are available in the main text.
